# Unraveling the interplay of kinesin-1, tau, and microtubules in neurodegeneration associated with Alzheimer’s disease

**DOI:** 10.3389/fncel.2024.1432002

**Published:** 2024-10-23

**Authors:** Siva Sundara Kumar Durairajan, Karthikeyan Selvarasu, Abhay Kumar Singh, Supriti Patnaik, Ashok Iyaswamy, Yogini Jaiswal, Leonard L. Williams, Jian-Dong Huang

**Affiliations:** ^1^Molecular Mycology and Neurodegenerative Disease Research Laboratory, Department of Microbiology, Central University of Tamil Nadu, Thiruvarur, India; ^2^Li Ka Shing Faculty of Medicine, School of Biomedical Sciences, The University of Hong Kong, Pokfulam, Hong Kong SAR, China; ^3^Mr. & Mrs. Ko Chi-Ming Centre for Parkinson’s Disease Research, School of Chinese Medicine, Hong Kong Baptist University, Hong Kong, Hong Kong SAR, China; ^4^Department of Biochemistry, Karpagam Academy of Higher Education, Coimbatore, India; ^5^Center for Excellence in Post-Harvest Technologies, North Carolina Agricultural and Technical State University, The North Carolina Research Campus, Kannapolis, NC, United States

**Keywords:** Alzheimer’s disease, axonal transport, molecular motors, kinesin I, microtubule, tau

## Abstract

Alzheimer’s disease (AD) is marked by the gradual and age-related deterioration of nerve cells in the central nervous system. The histopathological features observed in the brain affected by AD are the aberrant buildup of extracellular and intracellular amyloid-β and the formation of neurofibrillary tangles consisting of hyperphosphorylated tau protein. Axonal transport is a fundamental process for cargo movement along axons and relies on molecular motors like kinesins and dyneins. Kinesin’s responsibility for transporting crucial cargo within neurons implicates its dysfunction in the impaired axonal transport observed in AD. Impaired axonal transport and dysfunction of molecular motor proteins, along with dysregulated signaling pathways, contribute significantly to synaptic impairment and cognitive decline in AD. Dysregulation in tau, a microtubule-associated protein, emerges as a central player, destabilizing microtubules and disrupting the transport of kinesin-1. Kinesin-1 superfamily members, including kinesin family members 5A, 5B, and 5C, and the kinesin light chain, are intricately linked to AD pathology. However, inconsistencies in the abundance of kinesin family members in AD patients underline the necessity for further exploration into the mechanistic impact of these motor proteins on neurodegeneration and axonal transport disruptions across a spectrum of neurological conditions. This review underscores the significance of kinesin-1’s anterograde transport in AD. It emphasizes the need for investigations into the underlying mechanisms of the impact of motor protein across various neurological conditions. Despite current limitations in scientific literature, our study advocates for targeting kinesin and autophagy dysfunctions as promising avenues for novel therapeutic interventions and diagnostics in AD.

## Introduction

**FIGURE 1:**
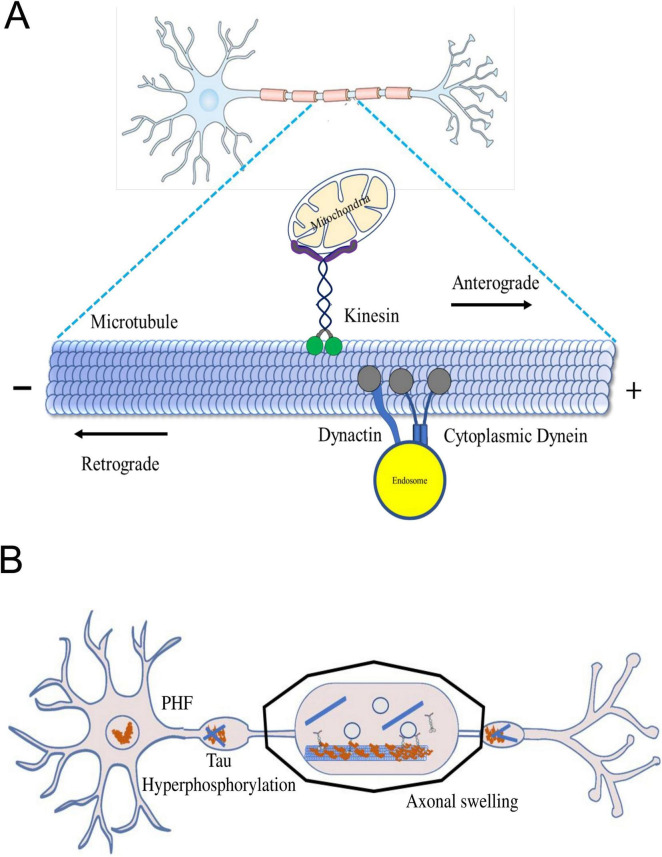
**(A)** The axonal transport machinery. Inside neurons, motor proteins like kinesin and dynein act as cellular couriers, shuttling different cargoes along microtubules. Kinesin moves cargo towards the axon tips, while dynein transports it back towards the cell body. This dynamic process ensures essential materials, organelles, and vesicles reach their destinations, maintaining neuron health. The microtubule network serves as a highway for these motor proteins, allowing precise and bidirectional cargo transport. **(B)** Axonal swelling in neurodegenerative diseases. Dysregulated cellular proteins cause harmful effects like neurotoxic peptides and inflammation. This leads to the formation of amyloid-β plaques, Tau hyperphosphorylation, and disrupted motor proteins, causing further damage to the brain. Stopping axon transport disrupts synapses and may lead to neurite issues.

Axonal transport plays a crucial role in anterograde transporting macromolecules and organelles from the cell body to the synapse. It also facilitates the retrograde transfer of signaling endosomes and autophagosomes for lysosome-mediated degradation. This complex mechanism hinges on two essential components: molecular motors responsible for propelling cargo and cytoskeletal elements that serve as tracks. Microtubules facilitate connecting motor proteins and their cargo, ensuring efficient transportation. Long-distance movement largely relies on microtubules, while actin filaments play a role in shorter-range transport. The microtubules within axons exhibit polarization, enabling directed movement by providing a structural orientation. The motor proteins involved in cargo transport are primarily kinesins and dyneins, with kinesins moving cargo anterogradely, away from the cell body, and dyneins handling retrograde transport toward the cell body (Maday et al., 2012). Microtubules comprising polarized tubulin polymers are characterized by their rapid-growing plus ends and more stable minus ends, which are arranged in a radial pattern within the soma, with their plus ends oriented toward the cortex (Stepanova et al., 2003; Figure 1). In dendrites, the arrangement of microtubules becomes more intricate, frequently forming arrays characterized by a combination of polarities (Baas et al., 1988; Kwan et al., 2008). However, when it comes to axons, the ends of microtubules appear to be capped by GTP (Bagdadi et al., 2024). The complex regulatory landscape points to an interplay of factors that govern the axonal transport dynamics.

Anterograde transport encompasses vital components for synaptic maintenance, cytoskeletal proteins crucial for axonal structure and function, and various proteins essential for neuronal activity, including mitochondria, chaperones, and glycolytic enzymes (Leopold et al., 1992; Elluru et al., 1995). In contrast, retrograde transport mainly involves endosomal/lysosomal organelles that convey degraded proteins to the cell body for degradation, along with neurotrophic factors vital for neuronal survival (Chevalier-Larsen and Holzbaur, 2006). The axonal transport system within nerve cells operates like a bustling highway, shuttling vital cargo to specific destinations. It functions at two main speeds: fast and slow. Fast axonal transport moves swiftly at 50–200 mm/day (equivalent to 0.5–3 μm/s), carrying essential items such as vesicles, organelles, and RNA, while slow axonal transport proceeds at a more leisurely pace of 0.1–3 mm/day. While some proteins are rapidly transported at speeds ranging from 50 to 200 mm per day (equivalent to 0.5–3 μm/s), rapidly reaching the axon’s tip, others exhibit significantly slower rates, approximately 0.1 to 8 mm per day (0.01–0.08 μm/s) (Lasek et al., 1984). In neurons, conventional kinesins are the primary type of molecular motors that move cellular organelles toward the plus end of microtubules (Case et al., 1997). Kinesins drive the anterograde fast axonal transport (FAT) of various membrane-bound organelles (MBOs), such as lysosomes and synaptic vesicles, to support cellular functions while also transporting axolemmal precursors to maintain the integrity of the axon’s membrane (Leopold et al., 1992). In contrast, retrograde FAT is performed by the complex motor protein cytoplasmic dynein (Susalka and Pfister, 2000). Axonal transport dysfunction has emerged as a significant factor in the pathogenesis of neurodegenerative disorders, with Alzheimer’s disease (AD) serving as a prominent case (Dan and Zhang, 2023). The protein tau, often associated with microtubules, has been reported to regulate axonal transport. Mutations in tau have been shown to destabilize microtubules, disrupting the transport process. The intricate process of FAT regulation involves multiple kinases that differentially modulate motor proteins. These kinases influence specific subunits within motor complexes, affecting their interactions with microtubules and ATPase activity (Zhang et al., 2012). Genetic mutations impacting molecular motor subunits operating along microtubules are linked to specific neuronal subtype degeneration (Shaw, 2005). The presence of abnormal vesicle accumulations and synaptic loss further emphasize the role of axonal transport deficits in AD pathology

This review focuses on the roles of kinesin-1, tau, and microtubules in the pathogenesis of AD. This demonstrates that kinesin-1 dysfunction, compounded by its interaction with hyperphosphorylated tau, significantly disrupts axonal transport, leading to synaptic impairment and cognitive decline. Also, genetic variations in kinesin family members, such as KIF5A and KIF5B, are implicated in AD progression by affecting axonal transport and mitochondrial mobility. It highlights the destabilizing effect of tau on microtubules, which further impairs kinesin-1’s transport function. Targeting kinesin-1 and autophagy dysfunctions could serve as promising therapeutic strategies for AD. Furthermore, we emphasize the necessity for continued exploration of the molecular mechanisms underlying kinesin-mediated transport in neurodegenerative diseases, as understanding these pathways could open new avenues for therapeutic interventions.

## Kinesin motor and Alzheimer’s disease: unraveling the connection

The kinesin family members are genetically associated with various human diseases, particularly the kinesin-1 superfamily constituents, including KIF5A, KIF5B, KIF5C, and the kinesin light chain (KLC), about AD Onset. Hares et al. (2017) demonstrated the activity of upregulated KIF5A expression at both mRNA and protein levels in AD human brain tissue (Hares et al., 2017). Similarly, associations are identified between heightened expression of KIF5A among AD patients with a concise period of disease progression (Kreft et al., 2014). In contrast, reduced KIF5A expression, associated with amyloid-beta (Aβ) toxicity, contributes to axonal mitochondrial transport defects in AD (Wang et al., 2019). Thus, protecting KIF5A function is proposed as a potential avenue to ameliorate mitochondrial abnormalities and treat AD (Wang et al., 2019). In cerebral ischemia, reducing KIF5B levels has been shown to protect neurons, as demonstrated in mice with a partial knockout of the KIF5B gene in stroke models. This protection works by regulating calcium influx from extrasynaptic N-methyl-D-aspartate receptors (NMDARs), thereby shielding neurons from NMDA-induced excitotoxicity and ischemic neurodegeneration. Consequently, inhibiting kinesin-1 function could potentially slow down the progression of neurodegeneration (Lin et al., 2019).

Zhao et al. reported that the targeted deletion of KIF5B results in decreased dendritic transport, diminished synaptic plasticity, and impaired memory (Zhao et al., 2020). Conditional knock-out mice of KIF5B show a substantial increase in dendritic spine removal compared to wild-type mice. Similarly, behavioral tests display shortfalls in synaptic plasticity, learning and memory. KIF5A and KIF5B serve distinct roles at excitatory synapses, with KIF5B knock-out mice exhibiting reduced synaptic transmission in CA1 hippocampal neurons compared to wild-type mice (Zhao et al., 2020). A study with mice lacking KIF5B found that completely removing KIF5B is lethal in defective early mouse embryo development (Tanaka et al., 1998). Although the knockout (KO) of KIF5B in a homozygous or conditional manner had an impact on brain development and memory, the authors of this article and other researchers have observed that heterozygous KIF5B KO mice (50% reduction) were healthy and functional without any neuroanatomical abnormalities (Lin et al., 2019; Zhao et al., 2020; Selvarasu et al., 2022). Our study indicates that reducing KIF5B, through knockdown or knockout, leads to less stable tau protein, which could cause it to aggregate or become dysfunctional. In contrast, increasing KIF5B via overexpression results in elevated tau protein levels, suggesting a regulatory role for KIF5B in maintaining tau stability and abundance, with possible implications for neuronal function or pathology (Selvarasu et al., 2022). Given that reducing KIF5B in P301S mice partially alleviates tauopathy, it is plausible to consider targeting KIF5B as a viable therapeutic approach for AD and other tauopathies (Selvarasu et al., 2022).

Dysregulated expression of KIF genes and genetic variations in the light chain shows susceptibility to AD. Hares et al. (2017) found that the mid-frontal cortex for both the gene and protein expressions of KLC1 and KIF5A were scrutinized in a study of AD-afflicted patient brain samples with susceptible genotypes. Intriguingly, the study unveiled connections between single nucleotide polymorphisms (SNPs) in the KIF5A gene, previously associated with susceptibility in multiple sclerosis and a decrease in KIF5A mRNA expression within the AD-affected cortex (Hares et al., 2019). The potential influence of genetic variations within the KIF5A gene locus contributes to disruptions in axonal transport, neuronal connectivity, and functional aspects of neurological conditions, including AD (Hares et al., 2019). The specific mechanisms underlying the influence of abundant KIF5A on hastened neurodegeneration within neurons necessitate further exploration. It remains unclear whether elevated KIF5B presence is the catalyst for, or outcome of, the accelerated neurodegenerative process. The vital role of KIF5A in upholding axonal transport introduces the intriguing concept that individuals with higher “reserves” of KIF5A might exhibit enhanced resilience against disruptions in axonal transport and subsequent pathological developments.

KIF5A and KIF1B play distinct roles in regulating mitochondrial mobility in rodents, with elevated KIF1B levels reducing mitochondrial transport and increased KIF5A expression enhancing mitochondrial mobility (Melo et al., 2013). In another study, Campbell et al. revealed that KIF5A cannot fully substitute for KIF1B in the transport of mitochondria. However, they also showed that KIF5A and KIF1B play dual roles in transporting non-mitochondrial cargoes, such as synaptic proteins, within peripheral sensory neurons. This investigation involved 47 AD brain samples and 37 control brain samples (Campbell et al., 2014). This finding suggests that these motor proteins have broader roles in neuronal function beyond merely influencing the transportation of mitochondria. Hares et al. (2017) evaluated the levels of KIF5A and KLC1 (mRNA relative to the neuronal density marker neuregulin). They observed a notable upregulation of KIF5A mRNA expression during Braak stages III-IV and V-VI (compared to stages I-II). The KLC1 mRNA levels exhibited a significant elevation in late Braak stage V-VI as opposed to the early stages, as assessed using the neurofibrillary tangle (NFT) scoring method (Braak and Braak, 1991). SNPs in KLC1 have no impact on KIF5A, while specific KIF5A SNPs *(rs12368653 and rs4646536)* are associated with decreased mRNA expression in AD. This trend was consistent at the protein level for KIF5A but not for KLC1 protein expression. These specific KIF5A SNPs play a significant role in reducing KIF5A mRNA expression in AD samples.

In an earlier study, Andersson et al. showed that KLC1 SNPs, including rs8702, may influence AD pathogenesis, especially in conjunction with APOE ε4, by affecting hyperphosphorylated tau levels in cerebrospinal fluid (Andersson et al., 2007). Consequently, Falzone et al. revealed that mice deficient in KLC show defects in axonal transport, resulting in tau hyperphosphorylation and NFT formation (Falzone et al., 2010). Morihara et al. examined 10 AD and 14 control brain samples, identifying a distinct splice variant of KLC1 known as variant E, revealing a notable increase in variant E levels in AD brains compared to normal controls. This KLC1 splice variant E was identified as an Aβ modifier and showed excess Aβ accumulation, suggesting that the elevated expression of this dysregulated KLC1 splice variant E may contribute to the development of AD (Morihara et al., 2014). These findings support the hypothesis that KLC-related disruptions in axonal transport may contribute to the pathological mechanisms underlying AD. Kreft and colleagues conducted a study involving 50 AD brain samples, revealing a five-fold increase in KIF21B protein levels in AD patients (Kreft et al., 2014). Notably, KIF21B expression levels were higher in Braak stage IV compared to stage V. The validation of other kinesins, including KIF5A, KIF5B, KIF5C, and KLC, has also linked them to AD. Specifically, KIF5A has shown abundant expression and is associated with the progression of AD (Kreft et al., 2014).

In a cohort study involving 12 AD cases and 12 healthy control samples, Chen et al. found a consistent reduction in KLC1 expression relative to normal KIF5A levels. These data indicates that there is a high probability of impaired axonal transport in neurons due to reduced KLC1 expression in AD. Such impairment could affect the efficient movement of cellular components along axons, potentially contributing to the pathophysiology of AD (Chen et al., 2021). All these clinical data provide valuable insight into the association between kinesin motor protein expression and AD. Kinesin family members, such as KIF5A, KIF5B, KIF5C, and KLC, are genetically associated with various human diseases, particularly AD. Elevated KIF5A expression is correlated with a shorter AD onset timeframe, observed alongside increased KIF5A mRNA and protein levels in AD brains. To fully understand kinesins’ role in AD, exploring the relationship between kinesin-1 and KLC expression with hyperphosphorylated tau and amyloid-β is crucial. It is still uncertain whether increased KIF5B levels trigger or result from the rapid neurodegeneration seen in the disease. KIF5A plays a vital role in maintaining axonal transport, suggesting it might possess increased resilience against disruptions in this process and the resulting pathological developments. However, inconsistencies in existing observations concerning AD hinder a definitive conclusion regarding whether alterations in KIF5 family members contribute to modifications in anterograde transport. These clinical studies underscore the need for additional statistical analysis and mechanistic studies to gain a deeper understanding of the role of kinesin in AD. These studies collectively suggest that variations in kinesin proteins, particularly in KIF5A and KLC1, could significantly impact AD development and progression.

## Neuronal transport and the role of microtubule-associated proteins

Microtubule-associated proteins, denoted as MAPs, are affixed along the length of axonal and dendritic microtubules. Their principal role involves facilitating the polymerization and reinforcement of microtubules, a task of particular importance due to the elevated levels of MAP expression within neurons. Microtubules typically exhibit greater stability within these cells than other cell types. MAPs have been shown to mediate interactions between molecular motors and microtubules, indicating their potential role as regulators of cellular transport processes (Vershinin et al., 2007; Dixit et al., 2008). MAPs play a role in stabilizing microtubule tracks, while motor proteins facilitate cargo movement along these tracks. Within the axon, microtubules create an unbroken array that stretches from the cell body to the growth cone at its farthest point. Proteins and membranous organelles are actively transported within axons along microtubules. The preferred orientation of each microtubule in this arrangement positions its growth-favored plus end away from the cell body (Baas et al., 1988). Motor proteins must maneuver through a densely populated microtubule framework adorned with non-motor MAPs. These MAPs play various roles in governing microtubule dynamics, stability, and turnover and also impact the transportation mediated by molecular motors (Subramanian et al., 2010). Tau, MAP2, or MAP4 are MAPs that adorn microtubules, aid in microtubule assembly, and modify microtubule physical characteristics (Nishida et al., 2023). The maintenance of axonal microtubule integrity is an important function of tau, the most abundant MAP in neurons. Furthermore, tau disrupts axonal neurites’ stability and the cytoskeleton’s composition, regardless of its capacity to bind to microtubules (Boumil et al., 2020). The hyperphosphorylated tau that is present abnormally in the AD brain is differentiated from transiently hyperphosphorylated tau by its capacity to capture normal tau, MAP1, and MAP2, thereby interfering with microtubule assembly (Durairajan et al., 2022). It can also form sedimentable cytosolic/oligomeric proteins that self-assemble into Paired helical filament (PHF) associated NFT. Dysregulation within the components of the transport apparatus, including microtubules, molecular motors and adaptors, has been identified as the underlying cause of various neurodevelopmental and neurodegenerative conditions (Sleigh et al., 2019; Berth and Lloyd, 2023). MAP hampers the movement of kinesin-1 both *in vivo* and *in vitro*, while its impact on dynein-driven motion is comparatively limited (Chaudhary et al., 2018). Furthermore, compromised axonal transport has been documented across a variety of neurological disorders.

## Kinesin motors: powering axonal intracellular movement

Kinesins are among the more prominent families of motor proteins. In the early 1980s, experiments in squid axoplasm uncovered an unidentified “translocator” responsible for axonal cargo transport (Allen et al., 1982; Brady et al., 1982). Vale et al. subsequently isolated a novel ATPase from the axoplasm and bovine brain, demonstrating its ability to move microtubules and transport beads along them (Vale et al., 1985). Kinesin marked the pioneering member of a diverse protein class (Hirokawa et al., 2009), essential for intracellular transport. Until now, 45 mammalian KIF genes have been recognized within the kinesin superfamily. This superfamily comprises 15 distinct kinesin families referred to as kinesin 1 to kinesin 14B. Each different kinesin is involved in different cargo molecule transport in different tissue-specific cell types (Miki et al., 2001). Apart from their function, kinesins were classified as N-terminal KIFs (N-KIFs), middle KIFs (M-KIFs), and C-terminal KIFs (C-KIFs) kinesins, the nomenclature being determined by the positioning of their motor domain (Aizawa et al., 1992; Lawrence et al., 2004). N-kinesins drive anterograde transport toward the plus end of microtubules (axon to dendrites). In contrast, C-kinesins drives retrograde transport toward the minus end (dendrites to axon) (Hirokawa et al., 2009). The motor activity displayed by KIFs is contingent upon the interplay between the motor domain and microtubule binding, with the energy needed for motion sourced from the hydrolysis of ATP (Hirokawa and Noda, 2008). The critical parts of major kinesin proteins include a motor section and a coiled-coil section.

**FIGURE 2:**
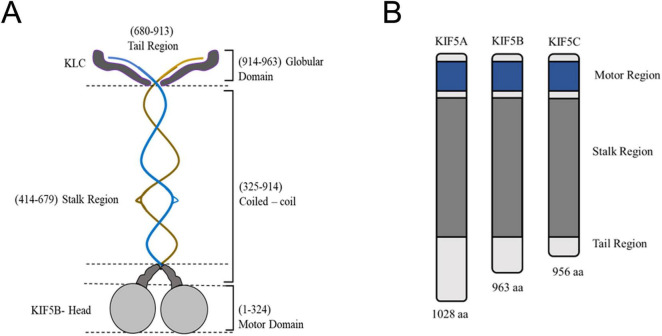
Structure of kinesin-1 and its domains. **(A)** The center blue and brown structure signifies the dimeric kinesin heavy chain, with each heavy chain comprising unique components: Motor Domain (Gray): Responsible for ATP and microtubule binding; Neck linker (Dark Gray): Experiences conformational alterations throughout the ATPase cycle. The stalk is interrupted by helical hinges in KIF5B, enabling the heads to swivel and the stalk to bend, resulting in a ‘folded’ conformation that is inactive when kinesin is at rest. The globular tail domain encompasses factors, such as the IAK motif, that affect the folding of kinesin into an inactive conformation. Additionally, the tail of the KIF5/kinesin-1 dimer binds to two light chains (KLC, approximately 570 residues in dark gray). The N-terminal residues of the light chains are involved in coiled-coil formation, linking the light chains to the heavy chains. Meanwhile, the C-terminal residues dock to the cargo or scaffolding proteins associated with the transport vesicle. The structural intricacies of kinesin, emphasize key features that contribute to its functionality in intracellular transport. **(B)** Three kinesin heavy chain isoforms exist based on tail variations. Although KIF5B is expressed ubiquitously, KIF5A and KIF5C are exclusively present in neurons.

Within the 14 families of kinesins, three main players drive axonal cargo transport: kinesin-1-4, with each’s specific role in axonal transport (Hirokawa et al., 2009). In vertebrates, kinesin-1 consists of two heavy chains (KHC) with motor and coiled-coil domains and two light chains (KLC) that attach to KHCs’ C-termini, aiding cargo binding (Vale, 2003) (Figure 2A). Four forms of KLC have been identified in vertebrates, including humans and mice. The conserved repeat in KLCs facilitates the recognition of MBO-associated cargo adaptor proteins. Notably, KIF5A, a member of the kinesin-1 family, is predominantly expressed in neurons, while KIF5B and KIF5C display widespread expression, with relatively elevated levels in neurons (Figure 2B) (Brady and Morfini, 2017; Antón et al., 2021). Of three KLC (1–3) genes, KLC1 and KLC2 are expressed in all tissues, and KLC3 is specifically expressed in the testis. Various combinations of heavy and light chains produce at least six different variants of kinesin-1. Various adaptor proteins assist kinesin-1, like Milton and Miro, for mitochondrial binding, unc-76/FEZ1 for presynaptic vesicles, and JNK interacting protein 1 (JIP1) for APP transport vesicles (Toda et al., 2008). Motor activity of KIF5 transporters is aided by several adaptor proteins like syntaxin 1 and syntabulin (Su et al., 2004). The KHC connects with syntabulin, facilitating the movement of vesicles containing amyloid precursor protein (APP), mitochondria, APOER2 and TrkB-containing vesicles, and presynaptic membrane vesicles along the axons (Huang et al., 2011).

KIF1A, KIF1Bα, KIF1Bβ, KIF3, KIF13, and KIF21B also play important roles in neuronal transport (Horiguchi et al., 2006; Kreft et al., 2014; Muhia et al., 2016). KIF1A is a motor protein primarily found in the brain and a solitary, non-associated form. KIF1A is responsible for transporting cargo vesicles containing membrane receptor precursors like synaptophysin, synaptotagmin, and syntaxin1A, all essential in neurotransmitter release (Guardia et al., 2016). KIF1B undergoes splicing to produce KIF1Bβ, a kinesin-3 family member involved in transporting synaptic vesicle precursors. KIF3, from the kinesin-2 family, aids in polarizing transport vesicles for neuronal growth, while KIF21B, a kinesin-4 family member, shows increased expression linked to neurodegenerative disease progression (Takeda et al., 2000; Kreft et al., 2014; Zhao et al., 2020).

## The kinesin-1 family in brain functions

KIF5 is the first conventional kinesin-1 group identified among the different kinesin groups, and it is the most abundant motor protein in neuronal cells (Hirokawa et al., 1989). Gene duplication processes result in three homologous KIF5 genes (KIF5A, KIF5B, and KIF5C) in vertebrates; however, in invertebrates like *Drosophila*, *C. elegans*, and *Aplysia*, there is only one KIF5 (Miki et al., 2001). Only a few studies have reported the functions of KIF5A, KIF5B, and KIF5C. The expression of KIF5A and KIF5C seems limited to neural tissues, in contrast with the ubiquitous expression of KIF5B (Navone et al., 1992; Niclas et al., 1994; Meng et al., 1997; Xia et al., 1998). The specific roles of KIF5A and KIF5C have not been determined, although it has been proposed that KIF5C plays a crucial role in the survival of motor neurons (Kanai et al., 2000). Mutations in KIF5A have been identified as the cause of a kind of hereditary spastic paraplegia in humans (Reid et al., 2002).

According to Tanaka et al. KIF5B-KO mice display aberrant mitochondrial location in their extraembryonic cells and are embryonic fatal (Tanaka et al., 1998). In KIF5B conditional knockout mice, dendritic spine morphogenesis, synaptic plasticity, and memory formation were impaired (Zhao et al., 2020; Fok et al., 2024). KIF5C-KO mice exhibit normal phenotypic characteristics; however, they display a reduced brain size and a relative decline in motor neurons compared to sensory neurons (Kanai et al., 2000). Neonatal KIF5A-KO mice are fatal, but their brains do not exhibit any noticeable histological abnormalities, except for the increased size of the nuclei and cell bodies of spinal cord motor neurons compared to wild-type mice (Xia et al., 2003). While the majority of conditional KIF5A-KO mice die within three weeks from seizures, a small percentage survive for three months or more and exhibit aberrant buildup of neurofilament (Xia et al., 2003). In cellular transport, functional redundancy is observed among KIF5A, KIF5B, and KIF5C proteins. Evidence demonstrates that exogenous expression of KIF5A or KIF5C can rescue impaired mitochondrial transport in KIF5B-lacking cells (Kanai et al., 2000). However, zebrafish studies reveal a specific role for KIF5A alone in the axonal transport of mitochondria, distinct from KIF5B and KIF5C (Campbell et al., 2014). This context-dependent specialization underscores the nuanced nature of molecular mechanisms governing intracellular transport.

## Interaction of kinesin with microtubules

The orientation of microtubules plays a crucial role in determining the direction of movement for kinesin-1 and other kinesins involved in cargo transport (Stenoien and Brady, 1997). Examination of kinesin-decorated microtubules with known polarity reveals that kinesin attaches to the plus end, which is formed by β tubulin, while at the minus end, it binds to the penultimate subunit, corresponding to α tubulin since the terminal subunit at the minus end is α tubulin (Fan and Amos, 2001). The main part of the kinesin motor domain is thought to be on β tubulin, but it is still debated to be possibly on α tubulin. Kinesin moves precisely in 8 nm steps on microtubules, and its compact motor domain is suitable for structural studies. Microtubules significantly boost kinesin’s activity, influencing its forward movement, though the exact binding sites are less understood than actin-myosin interactions (Woehlke et al., 1997; Song et al., 2001).

It is worth noting that posttranslational alterations of microtubules have demonstrated the potential to modulate the activity of molecular motors. A contentious discussion exists over the potential direct impact of α-tubulin K40 acetylation on enhancing the speed and affinity of kinesin-binding microtubules. Acetylation is a post-translational modification that distinguishes stable microtubules, primarily localized within axons. While some studies suggest increased acetylation enhances kinesin function, a recent study indicates it may not be the sole factor for its preference for axonal microtubules (Walter et al., 2012). The activity of molecular motors can be influenced by MAPs and the acetylation of α-tubulin at the K40 site within cells. Hammond et al. (2010) demonstrated that tubulin acetylation alone does not influence the velocity and run length of kinesin-1. This finding suggests that the acetylation state of microtubules alone may not be responsible for directing kinesin-1 toward axonal transport *in vivo*. Instead, a combination of tubulin acetylation with other posttranslational modifications or MAPs likely plays a role in guiding kinesin-1 *in vivo* (Hammond et al., 2010).

The protein KIF5, known for its tendency to accumulate in axonal microtubules, exhibits this behavior due to its affinity for binding to microtubules rich in GTP-tubulin. These microtubules contain specific regions where the plus-end-tracking protein End binding 1 (EB1) binds. Using the anti–GTP-tubulin antibody hMB11 to label these microtubules, researchers observed colocalization of KIF5 at the same attachment sites. Disrupting KIF5 accumulation at axon tips by blocking it with the hMB11 antibody suggests that GTP-tubulin levels guide the localization of KIF5 for vesicle transport within nerve cells. *In vitro* mobility assessments, KIF5 demonstrated a notable 30% increase in speed when moving along GTP-MTs compared to GDP-MTs (Vale et al., 1994). Although hybrid motor proteins could not definitively identify the precise signal, it strongly implies a potential role for GTP-MTs. Notably, Loop 11 of KIFs extends into a crevice between β-tubulin and α-tubulin (Song et al., 2001).

Within the mammalian brain, there are six distinct isotypes of β-tubulin, and class-III β-tubulin (TUBB3) exhibits a distinctive expression pattern exclusive to the nervous system. TUBB3’s expression is predominantly restricted to neurons (Katsetos et al., 2003), imparting significantly dynamic characteristics to microtubules compared to other β-tubulin isoforms (Panda et al., 1994). The interaction between TUBB3 and kinesin-1, particularly KIF5B, is crucial in axonal outgrowth. Studies suggest TUBB3 mutations can lower kinesin mobility and ATPase activity in KIF5B’s motor region. The vital role of the interaction between TUBB3 and kinesin-1 in axonal outgrowth is evident, as mutations in TUBB3 decrease kinesin motility and ATPase activity in KIF5B’s motor domain (Minoura et al., 2016). These intricate mechanisms underscore the underlying processes of intracellular transport within nerve cells.

## Kinesin-1 motor regulation: insights from head-tail interaction

Seeger and Rice explored the interaction between the tail of kinesin-1 and microtubules. They found that the kinesin-1 tail fragment strongly attaches to microtubules with high affinity, independently of the head domain (Seeger and Rice, 2010). This interaction overlaps with the binding site of tau, suggesting similarities to the consequences of MAP binding. The exact role of this strong kinesin tail-microtubule connection is not fully known. Still, its location near the tail’s conserved regulatory and cargo binding domains implies that it likely plays a crucial role in regulating kinesin. Also, the kinesin-1 tail fragment demonstrates a strong binding affinity to microtubules at a sub-micromolar level. This binding occurs independently of the head domain and shares a binding site with tau (Seeger and Rice, 2010). The interaction, similar to tau, is likely pivotal for kinesin regulation, given its proximity to the tail’s conserved regulatory and cargo-binding domains. Notably, the kinesin-1 tail and tau bind along the outer edge of the microtubule protofilament. While hTau40 features four consecutive microtubule-binding sites, the kinesin-1 tail, existing as a dimer, possesses two adjacent microtubule-binding sites on each heavy chain. This arrangement suggests the potential for two lateral contacts, bridging two protofilaments, in contrast to tau’s four longitudinal contacts along a single protofilament (Coy et al., 1999).

**FIGURE 3:**
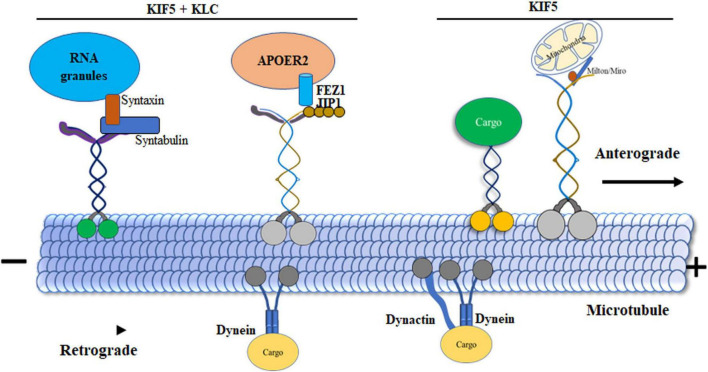
Dynamics of motor protein transport in microtubules. Kinesin-1 displays variable velocities during initiation, with the KIF5 tail domain forming a direct bond to cargos. KLCs act as connectors associating with cargos such as, c-Jun N-terminal kinase (JNK) interacting proteins (JIPs). Syntabulin, functioning as an adaptor protein, simultaneously binds to KIF5 motors and syntaxin 1. KLCs play a crucial role in linking vesicles containing receptors for apolipoprotein receptor 2 (ApoER2). JIP1 expedites the phosphorylation of JNK and facilitates the transport of APP. Adaptors Milton and Miro serve as bridges between KIF5 motors and mitochondria. Syntaxin 1, along with syntabulin and SNAP25 (RNA granules), directly binds to KIF5 motors, acting as adaptor proteins in the transport process. Additionally, Dynein is highlighted for its role in retrograde transport, moving organelles from presynaptic terminals to the cell body. This enhanced processivity is achieved through Dynein’s binding to the essential cofactor dynactin as an adaptor.

Unidirectional kinesins associated with microtubules that can move cargo vesicles over long distances help move the vesicles toward the cell’s periphery. Intracellular cargo is transported to the microtubule plus end by the motor protein kinesin-1, which needs energy from ATP hydrolysis. Depending on the stimulus, the cargo-moving protein kinesin-1 is turned on or off, facilitating accurate localization and delivery. In its folded shape, regulated kinesin-1 remains securely coupled to ADP, but has little microtubule binding. Despite the additional regulatory function provided by KLC, this folding can occur in their absence (Figure 3; Verhey et al., 1998; Cai et al., 2000). It is hypothesized that a direct connection between the head and tail of kinesin-1 controls the protein by blocking ADP release and halting its movement along microtubules (Friedman and Vale, 1999). The main function of regulation is to impede the productive interaction between the heads and microtubules. However, Dietrich et al. demonstrated a direct interaction between the inhibitory QIAKPIRP sequence in the tail domain and the Switch I region in the head of kinesin-1 (Dietrich et al., 2008). This finding suggests an additional regulatory mechanism by which the movement of the kinesin-1 tail on microtubules can be influenced. Specifically, this mechanism allows the kinesin-1 tail to temporarily pause its enzymatic activity while remaining bound to the microtubules. Thus, the interaction between the tail and the microtubule could halt the movement of the head. This occurs when the tail establishes a connection with the microtubule, causing the head to be immobilized. Simultaneously, the QIAKPIRP sequence of the tail interacts with Switch I, leading to the deactivation of the head. Short peptides with the QIAKPIRP sequence in the tail’s c-terminal part (residues 919–926 in human kinesin-1) have been shown to precisely inhibit kinesin-1 during the early step of ADP release induced by microtubules, contributing to autoinhibition and causing a paused state with high ADP affinity on microtubules (Hackney and Stock, 2000; Cai et al., 2007). It’s worth noting that this proposed “paused state” might seem counterintuitive to the primary role of tail-mediated regulation, which is to prevent kinesin-1 from binding to microtubules (Dietrich et al., 2008).

Kinesin-1’s core regulatory mechanism involves transitioning between a “folded” state and an “open” state. In the folded state, the tail’s conserved “QIAK” sequence binds to the heads, blocking ADP release and inhibiting ATPase activity (Hackney and Stock, 2000). In the open state, the heads become active when ATP replaces ADP, triggered by microtubule interaction. A cryo-electron microscope study revealed the tail’s simultaneous interaction with the head and microtubule, suggesting the formation of a complex where kinesin-1 might “park” as an enzymatically inactive motor tightly bound to microtubules. This behavior has been observed *in vitro*, but it’s *in vivo* purpose remains unknown. Studies in cultured cells and *in vivo* models have shown that kinesin-1’s c-terminal tail has an ATP-independent microtubule-binding site. Since full-length and truncated kinesin-1 heavy chains were observed to adorn microtubules in CV-1 cells where kinesin-1 was overexpressed (Navone et al., 1992), hypothesized that kinesin-1 may actively slide one microtubule against another via its head- and tail-binding sites. Further evidence suggests that kinesin-1 provides the force that drives the process of cytoplasmic streaming (Palacios and Johnston, 2002) can be seen in Drosophila oocytes, where arrays of microtubules cross-linked by kinesin-1 chain to disseminate yolk granules and other cytoplasmic components rapidly. Only the kinesin-1 heavy chain is required for this task, while the kinesin-1 light chain is unnecessary (Serbus et al., 2005).

In addition, Seeger and Rice (2010) found that the binding affinity of kinesin tail-microtubule interaction is very high. The kinesin tail binds tightly to microtubules, driven mainly by electrostatic forces, specifically at a site on alpha- and beta- tubulin known as helix 10 and sheet 9 (h10-s9), where numerous acidic residues are present. The kinesin-1 tail competes for this binding site with hTau40, and the interaction also involves helix 11 and helix 12 of tubulin in addition to the h10-s9 loop. Residues 892–914 within the kinesin-1 tail possess MAP-like properties for promoting microtubule assembly and stability (Seeger and Rice, 2010). Even though the tail exhibits a strong affinity for both KHC heads and microtubules, kinesin-1 is not perpetually locked in an inactive state nor constantly anchored to a microtubule transport state (Verhey et al., 1998; Cai et al., 2007; Dietrich et al., 2008). Although the kinesin-1 head and tail binding sites are distinct, their proximity suggests independent attachment to the microtubule.

According to Coy et al., cargo-less kinesin is present in a conformation where the tail is folded and compact, inhibiting ATPase activity in the motor domains (Coy et al., 1999). This mechanism is believed to be a preventive measure against many cellular consequences, including the ineffective depletion of ATP and the accumulation of kinesin proteins at the positive ends of microtubules. The binding of cargo to the tail domain of kinesin alleviates the inhibitory restriction imposed by the tail on the motor domain. This binding event induces a conformational change in kinesin, transforming it into an elongated state capable of ATP hydrolysis and propelling kinesin along microtubules. Therefore, the transition between these two forms of kinesin reflects a critical mechanism for controlling kinesin activity. The rationale for endorsing this model primarily relies on two pieces of evidence. Firstly, it has been observed that the tail of Drosophila kinesin-1 effectively suppresses the inherent ATPase activity of its motor domain, as demonstrated by Coy et al. and Hackney and Stock (Hackney and Stock, 2000). Secondly, RAN binding protein 2 (RANBP2), an external binding partner of kinesin-1, has been found to interact with KIF5B *in vivo* through its kinesin-binding domain (Cho et al., 2009). This interaction leads to an allosteric enhancement of KIF5B’s ATPase activity in the presence of microtubules. Previous studies have demonstrated that RANBP2 plays a crucial role in initiating and enhancing the functionality of KIF5B within a cell-free system with specific biochemical conditions (Cho et al., 2009).

Kinesin-1 movement on microtubules resembles the behavior of a “tether” when active motors are in a folded state. This configuration reduces the likelihood of cargo diffusing away. As the motor shifts from folded (inactive) to extended (active) form, its conformation shapes its movement on microtubules. It has been shown that truncated kinesin (kinesin-C), lacking its final 75 amino acids, exhibits a blend of processive and diffusive movement on microtubules (Lu et al., 2009). They also found that Kinesin-406, deficient in both the hinge and tail domain, can diffuse along the microtubule lattice with ADP present. This implies energy-saving diffusive behavior in folded kinesin, potentially aiding cargo engagement and obstacle handling. The kinesin motor, responsible for moving things inside cells, adjusts its activity and speed based on the availability of cargo and the cell’s conditions. The control of how fast it moves, its direction, and when it starts or stops involves complex mechanisms (Wong and Rice, 2010; Siddiqui and Straube, 2017; Nagpal et al., 2024).

## Kinesin, tau, and MAPs: the triad of neuronal transport

The microtubule network’s stability and spatial organization are governed by MAPs such as MAP2c and tau. Furthermore, the presence of MAPs on the surface of microtubules can disrupt the functioning of motor proteins that rely on microtubules for their movement (Seitz et al., 2002; Mandelkow et al., 2004). In a simplified kinesin-MAP interaction model, an increased level of MAP was found to reduce the run lengths and attachment frequency for kinesin. Seitz et al. examined the influence of neuronal MAPs on individual kinesin motor domains and fragments of the stalk domain. They found a decrease in the frequency of motor attachment due to MAP stimulation (Seitz et al., 2002). Once the kinesin motors successfully attached to the microtubules, they maintained a steady speed and run length.

The binding sites of tau on the surface of microtubules were revealed by using cryo-electron microscopy on unstained, vitrified specimens (Al-Bassam et al., 2002; Kar et al., 2003). The longitudinal binding of tau to the protofilament’s outer ridge was first reported by Al-Bassam et al. However, Kar et al. discovered that tau is attached to the microtubule’s inner surface (Kar et al., 2003). A study by Marx et al. addressed this ambiguity. It revealed that kinesin exhibits a strong affinity for tubulin, with tau molecules strategically positioned near tubulin, thus minimizing any potential interference between the motor protein and tau (Marx et al., 2005). The partial convergence of the binding sites elucidates the mechanism by which MAPs hinder the mobility of motor proteins in both cellular and laboratory settings. It was also postulated that the binding locations for MAP and kinesin on the surface of the protofilament would coincide. The findings suggest the potential for steric hindrance between the two molecular species when they compete for microtubule binding. In addition, the tail of kinesin also attaches to a distinct site on tubulin, which partially overlaps with the tau binding site (Seeger and Rice, 2010). Like how the tau protein interacts with α-tubulin, facilitating the assembly and stability of microtubules, the kinesin tail likewise enhances microtubule assembly and stability. There is competition between the kinesin tail and tau for binding sites on tubulin, and both contribute to microtubule stabilization, suggesting a potential role in regulating kinesin function (Seeger and Rice, 2010). Although the kinesin-1 head and tail binding sites are distinct, their close proximity suggests independent attachment to the microtubule.

**FIGURE 4:**
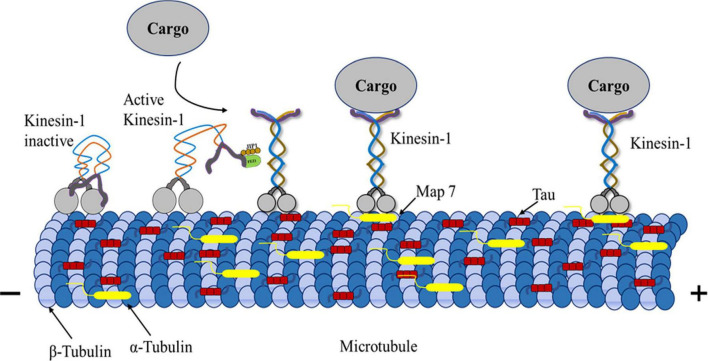
Kinesin-1, MAPs, microtubule triad Mechanism. Kinesin-1-driven transport can be negatively influenced by the MAPs. MAPs including tau, bind to microtubules’ outer surface, adversely affecting kinesin-1-driven transport. The kinesin motor domain interacts with β-tubulin, whereas tau interacts with α-tubulin. MAP7 interaction with kinesins promotes active motor movement in microtubules. Kinesin-tau interaction makes kinesin-1 motors and vesicles attach less or detach more during transport. Tau on microtubules regulates kinesin-1 transport efficiency by affecting how motor proteins interact with the tracks.

Cargos driven by kinesins and dyneins can rotate around microtubules to circumvent large obstacles. Maneuvering in a complex intracellular environment needs several parameters (Encalada and Goldstein, 2014; Fu et al., 2014). They are critical to regulating intracellular transport and maintenance of normal signaling and degradative pathways to prevent abnormal trafficking that is linked to developmental and neurological diseases (Hirokawa, 1998). After MTs are polymerized with tubulins, tau protein attaches to their inner surface, maintaining MT’s structural stability (Kar et al., 2003). In contrast, excess tau molecules added to stabilized MTs can adhere to their surface and obstruct kinesin migration (Hagiwara et al., 1994; Al-Bassam et al., 2002; Seitz et al., 2002). One possible means of regulating intracellular transport is the binding of MAPs along microtubules, which then modulates motor contact with the surface of the microtubules. The molecular mechanism of MAP-motor interference has been discussed (von Massow et al., 1989; Lopez and Sheetz, 1993; Hagiwara et al., 1994). The majority of research into how tau regulates cargo transport has focused on kinesin-1, with *in vitro* single-molecule experiments showing that tau attenuates kinesin-1 motility in a concentration-dependent manner (Vershinin et al., 2007; Dixit et al., 2008; McVicker et al., 2011; Kanaan et al., 2013). Research has shown that tau is crucial in controlling axonal transport by causing mitochondria and other vesicular cargoes to be mislocalized when overexpressed in neuronal and epithelial cells (Ebneth et al., 1998; Trinczek et al., 1999; Stamer et al., 2002; Soundararajan and Bullock, 2014). Because tau inhibits kinesin’s attachment to MTs but does not affect kinesin’s velocity, tau biases intracellular trafficking. As neurons rely on vesicles and organelles for cell activities, they are susceptible to oxidative stress due to lacking these components (Stamer et al., 2002). Similar results were seen with additional MAPs, such as MAP4 (Bulinski et al., 1997). However, how tau affects the motility of other kinesin family members is still unclear (Figure 4).

In contrast to kinesin-1 (KIF5B), kinesin-2 has an extended neck-linker region, facilitating maneuvering around obstacles like tau. The length of kinesin-2’s neck-linker plays a substantial role in determining kinesin’s ability to navigate microtubules, regardless of the presence or absence of tau isoforms such as 3-repeat short tau (3RS-tau) and 4-repeat long tau (4RL-tau) (Hoeprich et al., 2014). Moreover, kinesin-2 exhibits a shorter typical run length, resulting in fewer encounters with tau. The slower velocity of kinesin-2 may also allow it to wait for tau to clear its path during its processive movement along the surface of the microtubule. Additionally, Hoeprich et al. found that kinesin-2 employed a strategy of sidestepping to adjacent protofilaments to navigate around tau, and it demonstrated a higher frequency of switching between protofilaments compared to kinesin-1 (Hoeprich et al., 2017). The researchers attributed these characteristics to the longer neck linker in kinesin-2 compared to kinesin-1. In previous studies, it was shown that kinesin-8, possessing a neck-linker length of 17 amino acids similar to kinesin-2, exhibits a higher propensity to switch protofilaments compared to kinesin-1, which has a neck-linker length of 14 amino acids (Nitzsche et al., 2008; Bormuth et al., 2012). The study by Brunnbauer et al. provided evidence for protofilament switching due to elongating the neck-linker length of kinesin-1. The findings from these investigations combined indicate that the increased frequency of protofilament switching can be attributed to the longer neck linker of kinesin-2 (Brunnbauer et al., 2012).

Axonal microtubules become vulnerable to katanin and other microtubule-severing enzymes when tau is mislocalized during the beginning of AD (Qiang et al., 2006), which causes microtubule destabilization and axon degeneration. It is unclear what molecular mechanism tau uses to regulate. Regarding this, Siahann et al. demonstrated that tau molecules on microtubules interact to create cohesive islands, which differ in terms of kinetics from tau molecules that diffuse on microtubules singly (Siahaan et al., 2019). The tau islands serve as a protective barrier for microtubules against kinesin-1 motors and katanin. These islands possess unique regulatory properties that differ from a similarly dense layer of tau that is freely diffusing. Nevertheless, the kinesin-8 motors, which possess higher superprocessivity, can infiltrate the islands and induce their breakdown. Kinesin-8 remains bound even when a neighboring binding site on the microtubule is already occupied. Alternatively, the motor stops when encountering a bound tau molecule on the microtubule. It is then left to chance whether the next binding site becomes accessible when a tau microtubule-binding repeat temporarily unbinds. Then, kinesin-8 is strategically positioned to occupy a specific binding location on the microtubule, allowing it to systematically replace each of the microtubule-binding repetitions of a tau molecule. Superprocessive motors could control the location of tau in neurons in this way. Conversely, these motors can be controlled by tau islands, which are formed by traffic congestion at their boundaries and result in a decrease in their speed within the territories covered by these islands. Hinrichs et al. found that these cohesive areas are important for regulating cytoplasmic dynein and spastin (Hinrichs et al., 2012), and Tan et al. validated their existence (Tan et al., 2018). Therefore, these islands may serve as a measurement of post-translationally modified tubulin, making these areas selectively available to other MAPs. It is worth considering the intriguing prospect that impaired island assembly, which may be induced by hyperphosphorylation of tau, could potentially give rise to a multitude of subsequent pathophysiological effects in neurodegenerative diseases.

Unlike tau and MAP2 regulatory action on kinesin, MAP7 augmented the binding and processivity of kinesin-1 on microtubules, with its binding affinity enhanced by the localization of kinesin-1 in the C-terminal domain (Schroer, 2004; Magnani et al., 2007; Hooikaas et al., 2019). MAP7 proteins regulate kinesin-1’s microtubule activation and function (Hooikaas et al., 2019). In neurodegeneration, dysregulated tau disrupts the balance of motor transport toward plus and minus ends. The regulation of motor activities of kinesin-1, kinesin-2, and dynein by tau suggests that tau functions as an impediment on the microtubules, specifically hindering the processivity of kinesin-1. Single-molecule experiments reveal that tau exerts a stronger inhibitory effect on kinesin-1 compared to kinesin-2 and dynein, suggesting its potential role in shaping the spatial aspects of motor function and influencing the direction of intracellular cargo through distinct adjustments (Chaudhary et al., 2018). Tau’s impact on dynein’s retrograde transport is limited due to dynein’s reduced tau sensitivity. Tau moderately influences plus-end run length but increases long minus-end runs. Dynein and kinesin function as cellular cargo transporters, navigating opposite microtubule ends. Kinesin tends to detach tau patches from microtubules, while the dynein-dynactin complex often reverses direction or bypasses the movement. Significant inhibition of dynein was observed only at 10-fold of tau23 (a variant of tau) concentration, whereas the kinesin was detached at a one-fold increase of tau23 (Hagiwara et al., 1994; Dixit et al., 2008).

In neurons and other cell types, the dynactin complex facilitates the binding of the microtubule motor complex dynein to its cargoes (Schroer, 2004). The dynactin complex interacts with the tau protein at the N-terminal projection of tau through the N-terminal region of the p150 domain of the dynactin complex (Magnani et al., 2007). Tau and dynactin are highly colocalized, and tau facilitates dynactin complex adhesion to microtubules. Tau protein with frontotemporal dementia and parkinsonism linked to chromosome 17 (FTDP-17 ) mutations in the N-terminus of tau, which alters its binding to dynactin and causes an aberrant distribution of the protein in the axons of retinal ganglion cells in transgenic mice expressing human tau with a mutation in the microtubule-binding region. An understanding of the etiology of tauopathies from these results suggests a direct involvement of tau in axonal transport (Magnani et al., 2007).

The linker region between the KHC-interacting and cargo-binding domains of the tail helps partly inhibit its binding to microtubules. Despite the tail’s strong affinity for both KHC heads and microtubules, kinesin-1 is not permanently immobilized in an inactive state or constantly tethered to a microtubule in a non-transportable condition (DeLuca et al., 2001). While the kinesin-1 tail strongly adheres to KHC heads and microtubules, it doesn’t remain inactive or permanently bound to a non-transportable microtubule state. Within the KHC tail, a conserved region named the head-microtubule binding (HM) site serves a dual role in the regulation of kinesin-1’s motor activity. A Cryo-EM study revealed that the tail simultaneously interacts with KHC heads and microtubules. Notably, in kinesin-1’s self-restrained state, tails directly engage with KHC heads (Hackney and Stock, 2000; Cai et al., 2007; Dietrich et al., 2008). Tanaka et al. reported that abnormal clustering of mitochondria in the perinuclear region and embryonic lethality due to the results of the study indicated that the complete suppression of KIF5B or its heavy chain proved fatal, as KIF5B is one of the major motor proteins involved in cargo transport (Tanaka et al., 1998). The defective function of kinesin-1, overexpression of tau, and abnormal mitochondrial clustering collectively contribute to AD development.

## Impact of signaling pathways on kinesin-1 activity

Several studies postulate that the kinases and phosphatases involved in signal transduction also operate on the molecular motors that control the logistics of information transfer. Kinesins undergo phosphorylation by a partially shared group of serine/threonine kinases, and each phosphorylation event results in a distinct and specific outcome. The phosphorylation of the motor domain reduces motility, while the phosphorylation of the stalk and tail domains, depending on the residue and context, promotes cargo loading and unloading effects (Guo et al., 2020a,b). In addition, phosphorylation disrupts the interaction between accessory subunits and cargo proteins, as well as adaptor proteins and the motor. An early *in vitro* study revealed that protein kinase A (PKA), protein kinase C (PKC), and pp60c-src kinase could potentially phosphorylate bovine kinesin-1. PKC phosphorylated both KHC and KLCs, whereas PKA phosphorylated KLCs at several locations. The effect of kinesin motor domain phosphorylation by cJun N-terminal kinase -3 (JNK3) affected kinesin transport. K888, or full-length kinesin, and K432 (truncated kinesin) were used. Results from the JNK3, dynein, and microtubule-based single motility assay showed that Ser-175 (phosphomimic S175D) decreased the kinesin stall force. The modification of Ser-175 biased the transport toward the MT minus end direction for both kinesin and dynein in the MT (DeBerg et al., 2013).

## Kinesin-1 phosphorylation and its effects on motor function

Phosphorylation by PKA was found to enhance the motor function of purified kinesin-1 by increasing its microtubule-stimulated ATPase activity (Matthies et al., 1993). Further proof indicates that when the KHC motor domain is phosphorylated, it disrupts motor function. In one study, an overactive poly-Q expanded androgen receptor on spinal and bulbar muscular atrophy and, in turn, impaired kinesin-1 motility and axonal transport through phosphorylating KHC and activating JNK (Morfini et al., 2006). One role for kinesin-1 is to transport JIP family scaffolding proteins to sites where they can be in close contact with the JNK and the protein kinases (MKK4 and MKK7) that regulate through upstream signaling. As a consequence of this recruitment, JNK is able to activate via phosphorylating on threonine and tyrosine residues. Daire et al. postulated that the kinesin-1/JNK signaling pathway is an important regulator of microtubule dynamics in live cells and that it is necessary for cells to construct their interphase microtubule network in conjunction with the rescue factor CLIP-170 (Daire et al., 2009). They found that kinesin-1 and JNK promoted microtubule rescues to comparable degrees.

Likewise, a conserved serine residue (S175) of mouse KIF5A was phosphorylated when JNK3 was activated as a result of the generation of pathogenic huntingtin protein (Morfini et al., 2009b,2009a). The motor’s load-bearing capacity was diminished by phosphorylating the identical serine residue on KIF5B, leading to a preference for cargo transport toward the minus end while also preserving the autoinhibited state (Daire et al., 2009). Phosphorylation of S176 in mouse KIF5C potentially increased the ATPase rate but notably decreased microtubule binding in lab experiments, leading to a halt in kinesin-associated vesicle movement within axons (Padzik et al., 2016). These findings collectively suggest that phosphorylation at S175/176 stabilizes kinesin-1 in an autoinhibited state, reducing its load-bearing capacity without cargo *in vitro* and promoting vesicle movement toward the minus end *in vivo*. Similarly, KIF5A and KIFC play a role in regulating motor activity, but there was a functional redundancy between kinesin-1 KIF5A and KIF5C (Kanai et al., 2000). The KIF5-KLC complex, controlled by glycogen synthase kinase 3 (GSK3), governs fast anterograde axonal transport. Meanwhile, KIF5A is responsible for transporting postsynaptic density protein-95 (PSD-95) (Postsynaptic density protein-95) from presynaptic to postsynaptic dendrites, facilitating interaction with GABA_A_ receptors in inhibitory neuronal transmission (Morfini et al., 2002; Yoo et al., 2019).

In addition, Glycogen synthase kinase-3 beta (GSK3b), a tau kinase, could phosphorylate Drosophila KHC at S314, which is situated in the α6 helix bridging the head and neck-linker domain (Banerjee et al., 2021). Replacing S314 with phosphomimetic S314D or phosphodeficient S314A significantly impaired the motor’s performance, resulting in a marked decrease in ATPase activity and microtubule gliding in laboratory experiments (Banerjee et al., 2021). The neck-linker domain experiences substantial movement during the ATPase cycle, which generates force along microtubules (Rice et al., 1999; Vale and Milligan, 2000). This movement likely induces momentary tension on the α6 segment with each stepping cycle. Consequently, the phosphorylation of S314 can potentially modify helix arrangement and influence the overall dynamics of neck-linker movement (Qin et al., 2020). The gain and loss of function of KIF5B revealed that KIF5B is important for p38 MAPK kinase signaling (Yi et al., 2015). KIF5B and KIF13B transporter activity shows kinesin-1 (KIF5B) and KIF13B transport the small GTPase Rab6 exocytotic vesicles in MT plus end. Rab6 is the modulator that identifies the unfolded protein accumulation in AD (Elfrink et al., 2012). Knockdown of KIF5B/KIF13B in non-neuronal cells shows that KIF13B efficiently relocates the secretory vesicles from the KIF5B burden (Serra-Marques et al., 2020). Altogether, these findings point to separate pathways by which phosphorylation of several motor domain conserved serine residues may reduce kinesin-microtubule interaction and ATPase rate. Additional studies, including structure-function correlation, are required to determine the process and its importance *in vivo*.

## Kinesin-1 phosphorylation and its effects on cargo-motor interaction

It is well known that KHC phosphorylation impacts cargo association. According to Donelan et al., the glucose-dependent insulin release improved when KHC was dephosphorylated by protein phosphatase-2Bβ (PP2Bβ) in a Ca2+-dependent manner, whereas vesicle transport by kinesin-1 was decreased when KHC was phosphorylated in a casein kinase 2 (CK2) dependent manner (Donelan et al., 2002). Therefore, it was proposed that KHC dephosphorylation enhances insulin secretion in pancreatic β-cells upon insulin stimulation by relocating β-granules to the plasma membrane. The rapid axonal transport of membrane-bound organelles in squid axoplasm was hindered when CK2 was activated by Amyloid β (Aβ) oligomers in the axon, leading to an increase in KLC phosphorylation and the subsequent release of kinesin-1 from the membrane (Pigino et al., 2009; Schäfer et al., 2009). Based on these findings, phosphorylation of KHC and KLC is suggested to reduce cargo association and transit potentially. In line with this idea, it was discovered that cargo attachment was disrupted when KLC was phosphorylated by multiple kinases. Sato-Yoshitake et al. (1992) proposed that phosphorylation of KLC by PKA could decrease its affinity for isolated synaptic vesicles (Sato-Yoshitake et al., 1992)., likewise according to Morfini et al. Kinesin-1 was released from organelles attached to the membrane when KLC was phosphorylated by the GSK3b, which is abundant at the places where the neurites are produced (Morfini et al., 2002).

The lack of cargo and KLC phosphorylation can maintain kinesin-1’s autoinhibited conformation. Kinesin-1-dependent transport is activated and inactivated by KLC-KHC interaction, which is facilitated by the N-terminal coiled-coil domain of KLC (Gindhart et al., 1998; Friedman and Vale, 1999). Yip et al. found that KLC develops an autoinhibited folded conformation that involves the leucine-phenylalanine-arginine (LFR) motif and tetratricopeptide repeat (TPR) domains (Sato-Yoshitake et al., 1992). Being bound to KHC further solidifies the motor subunit’s fold-back inhibitory conformation, which is caused by the autoinhibited KLC. It has been proposed that the LFR motif may be disengaged by competing with the tryptophan-acidic (WD) motifs of the cargo/adaptor proteins. This would allow KHC to form an association with its positively charged tail domain, thus easing motor inhibition (Yip et al., 2016). Therefore, kinesin-1 might be restored to an inhibited state by phosphorylating the conserved TPR6 motif and the flexible C-terminal portion of KLC, which releases the cargoes/adaptors from the motor complex. Phosphorylation of kinesin-1 has dual effects on cargo and adaptor, potentially enhancing or inhibiting their formation. Despite the potential benefits of phosphorylating conserved serine residues in the N-terminal region of KLC and unknown KHC sites for adaptor binding and cargo transport, phosphorylating the C-terminal domain of KLCs disrupts cargo binding and stabilizes the motor’s autoinhibited conformation. The dual impact of kinesin-1 regulation and its role in intracellular transport processes warrants further investigation to uncover the underlying molecular mechanisms and physiological significance of the kinesin-1 regulatory process.

## Modulation of autophagy by kinesin-mediated transport

Cells employ macroautophagy (hereafter referred to as autophagy) and the ubiquitin-proteasome system (UPS) to eliminate damaged organelles and misfolded or aggregated proteins. Autophagy delivers cytoplasmic components to the lysosomes for degradation (Kaushik and Cuervo, 2015; Galluzzi and Green, 2019). In neurons, autophagy plays a crucial role in cellular homeostasis by facilitating the robust unidirectional retrograde trafficking of autophagosomes along axons. For two reasons, the accuracy of the autophagic process may be especially important in neurons. Neurons, being postmitotic, are particularly vulnerable to the harmful effects of misfolded proteins and organelles that build up over time. Furthermore, the spatial arrangement of neural processes can cause difficulties in effectively removing organelles and proteins through autophagy. Neurodegenerative diseases, including AD (Okamoto et al., 1991; Cataldo et al., 1996; Lee et al., 2011; Yang et al., 2017), PD, and others (Rubinsztein et al., 2005; Martinez-Vicente and Cuervo, 2007) are marked by the accumulation of autophagic vacuoles or autophagosomes, and there is evidence connecting autophagy abnormalities to these disorders. Upregulation of autophagy or blockage of autophagic flux and neural tissue-specific knockout of autophagy genes causes the indicative measure for neuronal dysfunctionality.

Extended microtubule-based retrograde transport of autophagosomes coincides with compartment maturation in the axon (Hollenbeck, 1993; Lee et al., 2011; Maday et al., 2012). Thus, axon autophagosome transit is spatially controlled. It is observed that, at first, the autophagosomes exhibit two-way movement at the distal end of axons in neurons and move inefficiently in both directions, likely due to both kinesin-1 and dynein motors. However, they soon adopt strong retrograde transport along the axon, driven by dynein and kinesin-1, while staying attached despite their predominant one-way motion, with few reversals or pauses (Maday et al., 2012). As part of the process of compartment maturation, autophagosomes travel great distances in the axon by retrograde transport supported by microtubules. The distal axon tip is the preferred location for autophagosome formation in dorsal ganglion neurons. After an ineffective bidirectional transport phase, the autophagosomes transition to unidirectional retrograde movement. According to Maday et al., autophagosomes in the mid-axon rarely show any pauses or direction changes (Maday et al., 2012). In cortical neurons, autophagosomes undergo dynein-dependent retrograde movement along the axon (Lee et al., 2011; Maday et al., 2012) discovered the surprising fact that dynein and kinesin stay linked to the axonal autophagosomes. In order to impose the dynein motor activity and minimize the movement of associated kinesins, autophagosomes must undergo strict regulation throughout their retrograde axonal trafficking. Therefore, controlling the retrograde axonal trafficking of autophagosomes is crucial for promoting dynein motor activity and suppressing the activity of related kinesins. Recently, our group was found that autophagy-mediated tau degradation in the cell soma may have occurred due to an increase in dynein-mediated autophagosome trafficking brought about by kinesin-1 loss (Selvarasu et al., 2022, 2024). The modulation of autophagy through kinesin-mediated transport is crucial for neuronal health, particularly in preventing neurodegenerative diseases like Alzheimer’s. Recent research highlights that reducing kinesin-1 activity can enhance dynein-driven retrograde transport, promoting efficient movement of both early and late endosomes by alleviating competition with opposing motors (Nagpal et al., 2024). Understanding the specific roles of kinesin and dynein in vesicular transport could lead to novel interventions for neurodegenerative conditions.

Autophagosomes merge with lysosomes or late endosomes when leaving the axon’s distal part, with complete acidification happening near the cell’s soma, highlighting their dependence on long-distance axonal transport for clearance mechanisms. The efficient recycling of amino acids for protein synthesis heavily relies on transporting autophagosomes to the cell soma. In addition, the significant retrograde motion observed during continuous autophagy in neurons helps maintain a balance in organelle and protein flow, countering their predominant outward movement through fast and slow anterograde transport processes (Maday and Holzbaur, 2014). JIP1 and huntingtin, as scaffold proteins, are involved in the regulation of autophagosome movement by interacting with both the kinesin-1 motor and the retrograde dynein-dynactin complex (Fu et al., 2014; Wong and Holzbaur, 2014). JIP1’s engagement with microtubule-associated protein 1A/1B-light chain 3 (LC3) plays a vital role in effectively restraining the activation of kinesin-1 on these cellular structures, resulting in the vigorous retrograde motion of autophagosomes within the axon. As autophagosomes traverse the axon, they undergo a maturation process, ultimately evolving into autolysosomes (Lee et al., 2011; Maday et al., 2012; Fu et al., 2014).

Upregulation of autophagy or blockage of autophagic flux and neural tissue-specific knockout of autophagy genes causes the indicative measure for neuronal dysfunctionality. Nutrient deprivation is the primary stimulus for autophagy in an *in vivo* setting. The absence of crucial nutrients can initiate autophagy. Kinesin-1 and JIP-1 are recruited to microtubules more effectively by hyperacetylation of tubulin, which also enables phosphorylation and activation of JNK. Additionally, JNK causes Beclin 1 to be released from Bcl-2-Beclin 1 complexes and recruited to microtubules, where it can potentially commence the generation of autophagosomes (Geeraert et al., 2010). Motor protein KIF5B has been shown to drive the formation of autolysosome tubulation (Du et al., 2016). Kinesin-1’s significance in autolysosome formation primarily hinges on its involvement in directing the positioning of autophagosomes and lysosomes. Multiple lysosomes fuse to form the autophagosome during the apex of autophagy, and KIF5B directly interacts with PtdIns (4,5)P2 in a clathrin-dependent manner and promotes the clustering of autolysosomes (Yu et al., 2010; Du et al., 2016). It has also been claimed that the enlistment of motor proteins to microtubules is enhanced by tubulin acetylation, leading to vesicular transport (Reed et al., 2006). KIF1A and KIF1Bβ have been reported to move late endosomes and lysosomes in non-neuronal cells (Matsushita et al., 2004; Bentley et al., 2015). Alternatively, lysosome transport in dendrites could be mediated by dynein (Wiggins et al., 2012). In autophagy-deficient cells, there is a pronounced accumulation of p62/SQSTM1, a selective autophagy receptor. It helps to recruit the ubiquitinated proteins and inclusion bodies to the autophagosome membrane (Bjørkøy et al., 2005; Pankiv et al., 2007). It has been confirmed that p62/SQSTM1 can significantly influence the efficiency of motor protein movement by preventing microtubule deacetylation and is selectively recognized and degraded through the autophagic pathway (Calderilla-Barbosa et al., 2014). MT-dependent transport mechanisms in neurons are increasing the recruitment of kinesin, dynein and dynactin to the more acetylated microtubules (Wang and Robbins, 2006; Dompierre et al., 2007; Nakai et al., 2007). Dynein transport could be disrupted in the absence of p62/SQSTM1. Recent research suggests that the regulation of autophagosome formation depends on maintaining a balance between dynein and kinesin activities (Cason and Holzbaur, 2023).

**FIGURE 5:**
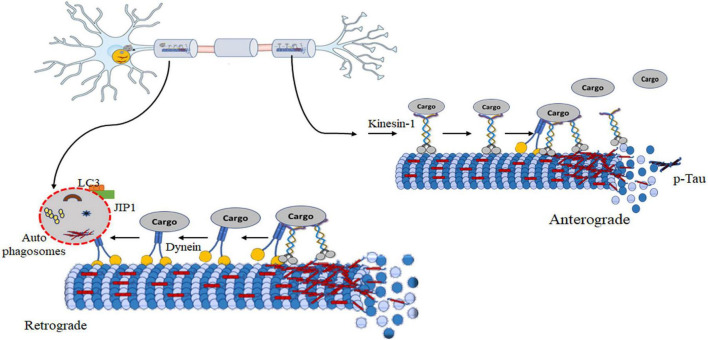
Model of autophagy regulation during axonal transport dysfunction induced by tau. Hyperphosphorylated tau disrupts kinesin-mediated anterograde transport. The formation of tau aggregates leads paired helical filaments and neurofibrillary tangles. These aggregates can sequester essential cellular components, including kinesin and its cargo, rendering them unavailable for transport. Targeting the removal of kinesin-1 is a possible way of reducing disease progression. It enhances dynein-mediated retrograde movement initiates autophagosome scaffold formation. Formation of autophagosomes is a critical step in autophagy for the cellular recycling process. It enables further transport or fusion with lysosome degradation of cellular components. Notably, targeting kinesin-1 removal presents a potential therapeutic avenue to modulate disease progression.

It has been revealed that depletion of KIF5B increases pericellular aggregation of autophagosomes and lysosomes in non-neuronal cells, suggesting that this KHC is essential for the peripheral mobility of these organelles (Cardoso et al., 2009). Remarkably, Geeraert et al. observed that starvation in HeLa cells leads to the suppression of centrifugal migration of autophagosomes through a kinesin-1-dependent mechanism (Geeraert et al., 2010). Our recent study found that tau transgenic mice (P301S Tau mice) had elevated levels of KIF5B in their brains. We also found that reducing or eliminating KIF5B expression significantly decreased tau levels in both animal and cell models of tauopathy (Selvarasu et al., 2022). We have recently demonstrated that tau binds to the microtubule-binding region of KIF5B and interacts with its motor domain. Inhibition of motor domain ATPase activity results from this interaction (Selvarasu et al., 2024). Consequently, the removal of KIF5B was found to increase autophagy, which, in turn, facilitates tau degradation (Figure 5). One possible mechanism for KIF5B-mediated tau degradation is that it interacts with tau, which in turn promotes autophagosome trafficking via dynein-dependent transport into autophagosomes, where tau is lysosomally degraded (Selvarasu et al., 2024).

## Conclusion

The interplay between kinesin motor proteins and MAPs and dysregulation within the transport apparatus plays a pivotal role in neuronal transport, impacting various neurodegenerative diseases, including AD. Clinical studies highlight the genetic association of kinesins like KIF5A, KIF5B, and KLC with AD progression, emphasizing the need for further meta-analysis. Post-translational modifications of kinesins, such as phosphorylation, influence kinesin binding and motor activity, emphasizing nuanced regulatory mechanisms within neuronal transport. The extensive repertoire of kinesin families, especially the KIF5 family (comprising KIF5A, KIF5B, and KIF5C), stand out as crucial players in anterograde transport, with disruptions leading to neurodegenerative conditions like Alzheimer’s and hereditary spastic paraplegia. Functional redundancy exists among KIF5s, yet their distinct roles in various tissues and developmental processes highlight the complexity of kinesin-mediated transport. MAPs like tau influence motor-protein interactions and microtubule stability, impacting intracellular transport. While Tau inhibits kinesin-1, kinesin-2 adapts with an extended neck linker. MAP7 enhances kinesin-1’s function, while MAP4 stabilizes microtubules.

Kinesin-1 regulation involves a delicate interplay between head-tail interactions, ATPase activity, phosphorylation and autoinhibition, ensuring controlled cellular transport. The diffusive movement of kinesin, especially in the presence of tau and while holding cargo, holds significance, potentially aiding in energy-efficient cargo engagement and obstacle handling during intracellular transport. In summary, kinesin-1 plays a vital role in cellular processes, particularly in phosphorylation dynamics and its links to neurodegenerative diseases. Its essential function in autophagy facilitates efficient intracellular transport and lysosomal degradation, highlighting its importance in maintaining cellular balance. By exploring the molecular mechanisms of kinesin-1 in autophagy, we can gain valuable insights into neurodegenerative disorders, opening up promising avenues for therapeutic interventions and underscoring its critical role in preserving cellular homeostasis.
